# Clinical outcome of subdural versus subgaleal drain after burr-hole drainage for chronic subdural hematoma

**DOI:** 10.1007/s00701-024-06320-7

**Published:** 2024-11-01

**Authors:** Sophie H. Carter, Maud J. de Rooij, Narjes Ahmadian, Anouk de Wit, Albert van der Zwan, Pierre A. J. T. Robe

**Affiliations:** Department of Neurosurgery, Utrecht University Medical Center, Utrecht, Netherlands

**Keywords:** Burr-hole drainage, Chronic subdural hematoma, Recurrence, Reoperation, Subdural drainage, Subgaleal drainage

## Abstract

**Background:**

Chronic subdural hematoma (CSDH) is commonly treated by burr-hole drainage with subgaleal or subdural drain insertion, mostly based on surgeon’s preference. We analyzed the recurrence rate and clinical outcomes after burr-hole drainage for CSDH and subdural or subgaleal drain insertion in a single center, retrospective cohort study.

**Methods:**

700 cases of burr-hole drainage for CSDH between 2017 and 2022 were included. Subdural drain insertion was compared to subgaleal drain insertion. The primary outcome were the rates of recurrence and reoperation. The secondary outcomes consisted of morbidity, postoperative complications, and mortality.

**Results:**

Baseline characteristics were comparable. The recurrence and reoperation rate after subdural drainage were respectively 15.3% (38/249) and 9.6% (24/249). The recurrence and reoperation rate after subgaleal drainage were respectively 13.4% (55/409) and 10.8% (44/409). There were no significant associations found in recurrence and reoperation rate between both drain insertions. No differences in morbidity, complication rate and mortality between drain insertion locations was found.

**Conclusion:**

We found relative equipoise between subdural or subgaleal drain insertion concerning recurrence, reoperation rate or clinical outcome. A large multicenter randomized controlled trial could be designed to further assess the outcomes of subdural and subgaleal drain placement after burr-hole drainage for CSDH.

**Supplementary Information:**

The online version contains supplementary material available at 10.1007/s00701-024-06320-7.

## Introduction

Chronic subdural hematoma (CSDH) is one of the most common neurosurgically treated diseases, characterized by a pathological accumulation of blood between the dura mater and the arachnoid mater. CSDH has an incidence of 8.2 to 48 per 100,000 persons per year [18, 20, 27], with a significantly increased occurrence in elderly with a tenfold higher risk in patients older than 70 in comparison to the general population [15, 24]. The incidence will continue to mount, as demography is showing a shift towards an aging population [16]. and increased use of anticoagulant therapy (ACT) or antithrombotic therapy (ATT) [3, 22]. In 5 to 30% of the chronic subdural hematomas a recurrence occurs, which can require re-operation [24, 28]. Reoperation leads to an increased risk for insults and infections, and is associated with recurrence of CSDH [18, 19].

Patients with a symptomatic and substantial chronic subdural hematoma are treated by neurosurgical evacuation, performed by burr-hole craniostomy or evacuation through craniotomy [30]. Since several studies have already shown a comparably low interventional risk and low risk of recurrence of CSDH [14, 28], evacuation through burr-hole craniostomy is considered the gold standard technique. The placement of a drain during the surgery results in lower recurrence rates and better outcome [1, 4, 23]. Such drains have been placed either in the subgaleal or in the subdural space, usually following surgeon’s preference [29]. Insertion of a subdural drain comes with a risk of complications such as bleeding, parenchymal brain injury, convulsion, and infections [1, 5, 17]. On the other hand, subgaleal drain insertion might in theory not be as effective as subdural drainage [10]. In the absence of evidence-based guidelines about the best location of the drain insertion, there remains a large practice variation in the choice of subdural or subgaleal drain insertion for the treatment of CSDH [6, 14].

Our center is also prone to such practice variation, based on surgeon’s theoretical preferences. This de facto leads to a pseudo randomization of the localization of drain placement based on the individual surgeon available at the time of CSDH surgical indication. This single center retrospective study investigates whether the location of drain insertion – either subdural or subgaleal – after burr-hole craniostomy for chronic subdural hematoma led to any difference in outcome.

## Methods

### Data collection

All adult patients (> 18 years old) who underwent burr-hole drainage for a de novo CSDH at our academic neurosurgical hospital, Utrecht University Medical Center, between 2017 and 2022, were retrospectively included. This study does not fall under the scope of the Dutch Medical Research Involving Human Subjects Act (WMO). It therefore does not require approval from an accredited medical ethics committee in the Netherlands. However, in the Utrecht University Medical Center, an independent quality check has been carried out to ensure compliance with legislation and regulations (regarding Informed Consent procedure, data management, privacy aspects and legal aspects). Patients who underwent a craniotomy for CSDH were excluded. All patients were included per hemisphere case to avoid misrepresentation in patients with a bilaterally present CSDH who would be treated unilaterally or differently on both sides. If CSDH was present bilaterally but only treated with burr-hole drainage unilaterally this was thus included as unilateral hematoma. Surgical indications for CSDH were performed by the surgeon on duty. No patient was treated with embolization at our center. Standard treatment for chronic (i.e. liquified) hematoma at our center is burr hole craniotomy. Obviously, multiloqulated hematoma with thick membranes, are sometimes treated with craniotomy and were excluded from this series. Likewise, patients (*n* = 3) who were considered for burr hole drainage but were converted to mini craniotomy during the procedure due to poor drainage, were also excluded.

Baseline characteristics included are sex, age, smoking, medical history affecting bleeding risk, including diabetes, kidney failure and liver disease, use of ACT/ATT and whether its management around surgery is according to our institutional protocol (Table 1) or had to be stopped faster than advocated because of (relative) emergency. The condition of the patient at admission, including modified Rankin Scale (mRS) and symptoms was also recorded. The following procedural details were included: laterality, number of burr-holes, anesthesia type, drain type and duration of drainage in days. The follow-up period was six weeks, during which the postoperative assessment was conducted by the surgeon at our center. Subsequent follow-up was then scheduled with the neurologist at the secondary center. In our center it is not standard care to perform a postoperative computed tomography (CT) scan. This was only performed on clinical suspicion of recurrence.

**Table 1:** Institutional ACT/ATT discontinuation protocol

	Preoperative time of discontinuation
Acetylsalicylic acid	−
Carbaselatecalcium	5 days
Clopidogrel	5 days
Ticagrelor	3 days
Fenprocoumon	5 days
Acenocoumarol	3 days
DOAC	2 days

*ACT* anticoagulant therapy

*ATT* antithrombotic therapy

*DOAC* direct oral anticoagulant

Primary outcome measure was rate of recurrence, which was defined as ipsilateral reaccumulation of CSDH on CT-scan with neurological symptoms within six months after primary surgery. The time of recurrence was assessed, distinguishing between spontaneous and traumatic recurrences (i.e. due to a new trauma), as well as evaluating the need for reoperation. Secondary outcome measures included assessing morbidity and mortality after the surgery. Not all complications were attributed to the laterality but rather to patient specific factors, such as deliria or systemic infection. Therefore, morbidity and mortality were assessed per patient, instead of per hemisphere. Morbidity was evaluated by mRS score after surgery at the six-week postoperative appointment, and postoperative complications, including bleeding, infarction, convulsion, wound leakage, wound infection, hematoma infection or empyema, suturing of drain, systemic complications such as deliria, pneumonia, or urinary tract infection and mortality as death within six weeks after surgery.

### Operative procedure

Burr-hole drainage was performed after recent CT determined CSDH with hypodense components and significant symptoms under general anesthesia, unless perioperative anesthesia risk was considered to be too high. Patients were positioned supine, shaved, marked, disinfected, and draped. After incision (generally on Kochers point and parietal, depending on the maximum thickness of the hematoma), two burr-holes were created. According to institutional standard, generally two burr-holes were drilled, unless the surgeon deemed that one would be enough based on the characteristics of the CSDH. The dura was coagulated and opened, and the hematoma was drained, followed by irrigation with physiological salt solution. Next, a subdural or subgaleal drain was inserted by surgeon’s preference, Neuromedex Bactiseal external ventricular drain or a NeuroVac Silikon-Jackson Pratt. The drain was then tunneled subcutaneously away from the incision and anchored with a staple or suture to the scalp. The incision was closed in layers with sutures or, in rare cases, with staples. Postoperatively, patients were usually sent to the neurosurgical ward and occasionally to the neurosurgical medium care or intensive care unit. Flat bedrest was ordered while the drain was in situ. Drains were removed when not productive. After drain removal and clinical improvement, patients were discharged or transferred to secondary centers for further recovery. If allowed by clinical condition, ACT/ATT was restarted five days after surgery.

### Statistical analysis

Statistical analysis was performed using IBM SPSS Statistics version 26 [12]. For all outcome measures Pearson Chi-squared test or independent t-test was used to compare both cohorts, calculating odds ratios (OR) with 95% confidence intervals (CI). Multivariable logistic regression analyses were used to assess predictors of potential confounders, namely sex, age, the number of burr-holes, drain duration of more than one day, use of ACT/ATT and medical history, calculating adjusted OR with 95% CI. Because the secondary outcome measures were calculated per patient instead of per hemisphere, laterality was included in multivariable logistic regression. A *p* value of < 0.05 was considered statistically significant.

## Results

### Participants

**Fig. 1:**
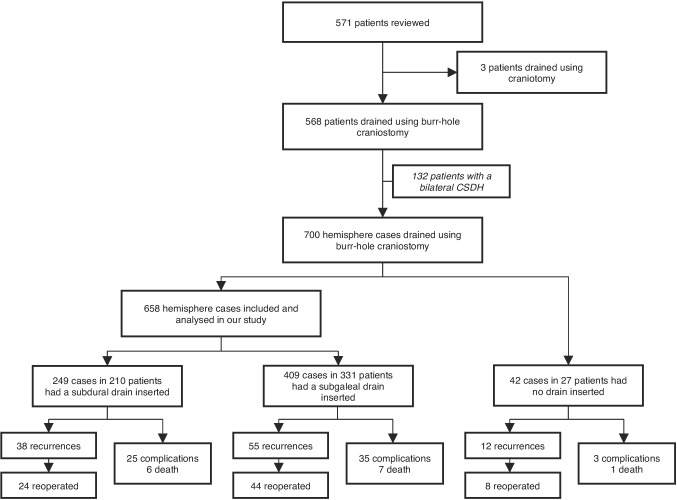
Data collection profile and outcome flowchart

Data from 571 patients were reviewed, out of which 568 underwent burr-hole craniostomy. Among these, 132 patients had bilateral hematoma, resulting in a total of 700 CSDH cases. A drain was inserted in 658 of these (Fig. 1). In few cases (*n* = 42) no drain was inserted based on surgeons impression that the brain veered back to the convexity completely and no subsequent drainage was deemed necessary. Demographics, admission status details, operative techniques and postoperative care are shown in Table 2.

**Table 2:** Baseline characteristics of subdural and subgaleal drain groups

	Subdural drain	Subgaleal drain	*p* value
Age, mean (SD)	75.9 (± 11.1)	74.6 (± 10.6)	0.442
Sex			0.258
Male	175/249 (70.3)	304/409 (74.3)	
Female	74/249 (29.7)	105/409 (25.7)	
Smoking*	9/86 (10.5)	29/165 (17.6)	0.136
Medical history			
Diabetes*	41/248 (16.5)	69/409 (16.9)	0.910
Kidney failure*	16/248 (6.5)	14/409 (3.4)	0.071
Liver disease	8/249 (3.2)	11/409 (2.7)	0.697
Use of ACT/ATT			0.064
None	116/249 (46.6)	221/409 (54.0)	
Yes	133/249 (53.4)	188/409 (46.0)	
Quit according to protocol (Table 1)*	92/131 (70.2)	153/186 (82.3)	**0.012**
Restarted after 5 days or more*	99/105 (94.3)	143/149 (96.0)	0.532
Admission status			
Modified Ranking Scale score			0.663
good 0–1	0/249 (0.0)	0/409 (0.0)	
medium 2–3	98/249 (39.4)	168/409 (41.1)	
poor > 4	151/249 (60.6)	241/409 (58.9)	
Symptoms			
Sensomotoric	115/249 (46.2)	212/409 (51.8)	0.160
Cognitive	144/249 (57.8)	233/409 (57.0)	0.828
Gait disturbance/ataxia	109/249 (43.8)	190/409 (46.5)	0.503
Headache	75/249 (30.1)	123/409 (30.1)	0.990
Convulsion	5/249 (2.0)	5/409 (1.2)	0.424
Operative technique			
Laterality of chronic subdural hematoma**			0.464
Bilateral	42/210 (20.0)	75/331 (22.7)	
Unilateral	168/210 (80.0)	256/331 (77.3)	
Number of unilateral burr-holes			0.078
One	49/249 (19.7)	59/409 (14.4)	
Two	200/249 (80.3)	350/409 (85.6)	
Type of anesthesia			**0.009**
General	239/249 (96.0)	405/409 (99.0)	
Local	10/249 (4.0)	4/409 (1.0)	
Postoperative care			
Duration of drain placement			**0.003**
One day or less	184/249 (73.9)	342/409 (83.6)	
More than one day	65/249 (26.1)	67/409 (16.4)	
Postoperative CT scan*			0.923
None	143/247 (57.9)	237/409 (57.9)	
During admission	41/247 (16.6)	72/409 (17.6)	
After discharge	63/247 (25.5)	124/409 (24.4)	

All data presented as n/N (%)

Bold values are statistically significant (*p* < 0.05)

*SD* standard deviation

*ACT* anticoagulant therapy

*ATT* antithrombotic therapy

*CT* computed tomography

*missing data

**analysed per patient, not per hemisphere

### Descriptives

Of the 658 cases that received perioperative drainage, a subdural drain (SDD) was inserted in 249 hemispheres (37.8%), while a subgaleal drain (SGD) was used for drainage in 409 hemispheres (62.2%). In the SDD group 53.4% (133/249) used ACT/ATT, this was 46.0% (188/409) for the SGD group (*p* = 0.064). The ACT/ATT of those patients could be tapered down according to the institutional protocol in 70.2% of the CSDH in the SDD group (92/131), versus 82.3% (153/186) in the SGD group (*p* = 0.012). In the SDD group, 239 cases (96.0%) were performed under general anesthesia, while in the SGD group, 405 cases (99.0%) received general anesthesia (*p* = 0.009). Drain duration in the SDD group was significantly longer than in cases with a SGD inserted (*p* = 0.003). All other baseline characteristics were comparable between both drain groups.

The no drain group (NDG) scored significantly (*p* = 0.010) poorer on mRS at admission compared to the drain group (DG), respectively 81.0% (34/42) and 59.6% (392/658).

### Outcomes

#### Overview of all included cases

The overall recurrence rate in the cohort of 658 cases receiving a drain was 14.1%. The recurrence rate of 15.3% (38/249) in the SDD group was similar to that in the SGD group (13.4%; *p* = 0.517). The overall reoperation rate was 10.3%. The rate of reoperation was 9.6% (24/249) in the SDD group. This does not significantly differ from 10.8% (44/409) in the SGD group (*p* = 0.647). In three cases bilateral hematoma was operated on unilaterally, none of these cases had a recurrence on either side. Results on recurrence rates are shown in Table 3.

**Table 3:** Comparison of recurrence between subdural and subgaleal drain groups

	Subdural drain	Subgaleal drain	Odds Ratio (95% CI)	*p* value
Recurrence	38/249 (15.3)	55/409 (13.4)	0.86 (0.55–1.35)	0.517
Cause of recurrence				
Spontaneous	37/38 (97.4)	49/55 (89.1)	0.22 (0.03–1.91)	0.137
Traumatic	1/38 (2.6)	6/55 (10.9)	4.53 (0.52–39.27)	0.137
Time of recurrence in weeks, mean (SD)	4.21 (± 3.53)	4.67 (± 3.28)	NA	0.730
Reoperation	24/249 (9.6)	44/409 (10.8)	1.13 (0.67–1.91)	0.647

All data presented as n/N (%)

Bold values are statistically significant (*p* < 0.05)

*CI* confidence interval

*SD* standard deviation

*NA* not applicable

Subanalysis of recurrence and reoperation rates for patients treated with single burr hole drainage are presented in supplementary data. No significant difference was found.

Clinical outcomes are shown in Table 4. Six weeks mortality occurred in six patients of the SDD group (0,03%), in seven patients in the SGD group (0,02%) and in one patient in the NDG group (0,04%). In five patients the cause of death was pre-existent morbidity, in three patients infarction, in two patients pneumonia, in two patients empyema, in two patients acute subdural hematoma, one of which immediately followed the removal of a subdural drain.

**Table 4:** Comparison of clinical outcomes between subdural and subgaleal drain groups

	Subdural drain	Subgaleal drain	Odds Ratio (95% CI)	*p* value
Postoperative complications	25/210 (11.9)	35/331 (10.6)	0.88 (0.51–1.51)	0.631
Bleeding	1/210 (0.5)	6/331 (1.8)	3.86 (0.46–32.28)	0.180
Infarction	1/210 (0.5)	4/331 (1.2)	2.56 (0.28–23.03)	0.386
Convulsion	8/210 (3.8)	5/331 (1.5)	0.39 (0.13–1.20)	0.089
Woundinfection, leakage	3/210 (1.4)	6/331 (1.8)	1.27 (0.32–5.15)	0.730
Infected hematoma/empyema	3/210 (1.4)	1/331 (0.3)	0.21 (0.02–2.02)	0.136
Drain sutured	1/210 (0.5)	1/331 (0.3)	0.63 (0.04–10.18)	0.745
Systemic complications	7/210 (3.3)	12/331 (3.6)	1.09 (0.42–2.82)	0.857
Death	6/210 (2.9)	7/331 (2.1)	0.74 (0.24–2.21)	0.583
mRS score after surgery				0.296
good 0–1	126/210 (60.0)	177/331 (53.5)	0.77 (0.54–1.09)	0.136
medium 2–3	73/210 (34.8)	137/331 (41.4)	1.33 (0.93–1.90)	0.123
poor > 4	11/210 (5.2)	17/331 (5.1)	0.98 (0.45–2.13)	0.958

All data presented as n/N (%)

Bold values are statistically significant (*p* < 0.05)

*CI* confidence interval

*mRS* modified Rankin Scale

An analysis comparing the cohort of cases receiving a drain (drain group; DG) with cases without drain showed a significantly higher recurrence rate of 28.6% (12/42; OR 0.41; 95% CI 0.20–0.83; *p* = 0.020) in the NDG. However, the reoperation rate of 19.0% (8/42) in the NDG was not significantly different from the DG (OR 0.490; 95% CI 0.22–1.10; *p* = 0.133).

#### Recurrence of CSDH

Potential predictors of recurrence were tested in a multivariable logistic regression model together with type of drain insertion (subdural drain versus subgaleal drain). On multivariable analysis, presence of liver disease (*p* = 0.001) was significantly increasing the risk of recurrence with an OR of 4.96 (95% CI 1.91–12.90). The other covariables and drain type were not found to be a predictor of recurrence in the multivariable analysis. Univariable sub analysis showed no significant influence of ACT/ATT use, nor whether it was tapered down according to protocol, on recurrence rate.

#### Reoperation of CSDH

Multivariable analysis on reoperation rate shows no significant difference between both drain groups. Merely presence of liver disease is found a significant predictor of reoperation (*p* = 0.027) with an OR of 3.35 (95% CI 1.15–9.78). Results of all multivariable analyses are presented in Table 5. ACT/ATT use and whether it was tapered down conform protocol was not found significant on reoperation rate.

**Table 5:** Multivariable logistic regression for predictors of recurrence and reoperation

	Recurrence		Reoperation	
	Odds Ratio (95% CI)	*p* value	Odds Ratio (95% CI)	*p* value
Subgaleal drain (reference subdural drain)	0.89 (0.56–1.41)	0.609	1.16 (0.68–1.99)	0.595
Two burr-holes (reference one burr-hole)	1.32 (0.68–2.54)	0.413	1.65 (0.72–3.74)	0.235
Drain duration more than one day (reference one day or less)	1.06 (0.61–1.85)	0.825	1.15 (0.62–2.13)	0.668
Use of ACT/ATT	1.32 (0.82–2.13)	0.252	1.00 (0.59–1.72)	0.991
Female (reference male)	0.66 (0.39–1.15)	0.142	0.69 (0.37–1.28)	0.239
Age	1.00 (0.98–1.03)	0.733	1.02 (0.99–1.04)	0.282
Medical history				
Diabetes	1.15 (0.65–2.04)	0.625	1.06 (0.55–2.05)	0.864
Kidneyfailure	1.72 (0.70–4.24)	0.240	1.67 (0.60–4.66)	0.325
Liver disease	4.96 (1.91–12.90)	**0.001**	3.35 (1.15–9.78)	**0.027**

Bold values are statistically significant (*p* < 0.05)

*CI* confidende interval

*ACT* anticoagulent therapy

*ATT* antithrombotic therapy

#### Postoperative complications after burr-hole craniostomy

Covariables on increasing the risk of postoperative complications and type of drain insertion (SDD versus SGD) were analyzed in a multivariable logistic regression model. Only preoperative use of ACT/ATT remained statistically significant on complication rate (*p* = 0.001; OR 2.89; 95% CI 1.52–5.49). The other analyzed covariables and type of drain were not found to be predictive factors for postoperative complications (Table 6).

**Table 6:** Multivariable logistic regression for predictors of postoperative complications

	Odds Ratio (95% CI)	*p* value
Subgaleal drain (reference subdural drain)	0.93 (0.52–1.65)	0.802
Two burr-holes (reference one burr-hole)	0.80 (0.37–1.71)	0.560
Drain duration more than one day (reference one day or less)	0.87 (0.43–1.78)	0.705
Use of ACT/ATT	2.89 (1.52–5.49)	**0.001**
Female (reference male)	0.93 (0.49–1.79)	0.837
Age	0.98 (0.95–1.01)	0.147
Medical history		
Diabetes	0.74 (0.34–1.61)	0.451
Kidneyfailure	2.41 (0.88–6.56)	0.086
Liver disease	1.65 (0.35–7.81)	0.530
Bilateral hematoma	1.22 (0.62–2.41)	0.569

## Discussion

### Principal findings

In this retrospective study, no significant difference was found in the recurrence rate between the insertion of a subdural or subgaleal drain after burr-hole craniostomy for chronic subdural hematoma. Similarly, the location of drain insertion did not significantly affect the reoperation rate. However, due to the small number of reoperation cases, interpreting these findings becomes challenging. Presence of liver disease was identified as predictor of both recurrence and reoperation, however, with wide confidence intervals. No significant differences were found between the two drain types in relation to clinical outcome. Notably, the use of ACT/ATT was found to be predictor of postoperative complications, making a significant contribution to the overall complication rate. Similar to the results of Santarius et al. [23], we found that not inserting a drain results in a significant higher risk of recurrence. Recurrences within six months were reported, there were no cases excluded due to this limitation.

### Comparison with existing literature

The findings of our study are consistent with the literature, showing no evidence for a significant difference in recurrence rate based on location of drain insertion [2, 7–9, 11, 23, 26, 30, 31]. Our study demonstrates a recurrence rate similar to what has been reported in the literature, within the range of 5–30% [24, 28] According to a meta-analysis conducted by Henry et al. [11], the recurrence rate after SDD of CSDH was found to be 11.2%, and SGD 8.3%. In our study, we observed a subdural and subgaleal recurrence rate of respectively 15.3% and 13.4%. Initially it seems that our reported recurrence rates for both drain insertions are slightly higher than those reported by others. However, all studies, except for Zumofen et al. [26], define recurrence as a recurrence for which reoperation is needed [7–9, 11, 23, 26, 30], not as ipsilateral reaccumulation with neurological symptoms, as we do. Ergo, it is more appropriate to compare our reoperation rates of 9.6% after SDD and 10.8% after SGD insertion to the percentages reported before, then being very similar.

In agreement with existing literature [1, 4, 23], we found a significant lower recurrence rate after insertion of a drain compared to no drain, however the reoperation rate was not significantly different. At admission the NDG was in poorer condition, which might have led to the surgeon’s choice to withhold drain insertion. This initial poor preoperative condition could also affect the decision to refrain from reoperation.

While no statistical significance was observed in clinical outcome, many patients were improved following surgery (good mRS scores of 0–1 preoperatively 0%, postoperatively 60%). The clinical outcomes were also evaluated in several studies. Zhang et al. [30], Chih et al. [7] and Gazzeri et al. [8] reported no significant associations with functional outcomes. Kaliaperumal et al. [13] reported better functional outcomes in the subgaleal drain group at 6-month follow-up, but this may be influenced by initial differences in preoperative mRS scores. Singh et al. [25] found a higher incidence of postoperative seizures in the subdural drain group. Oral et al. [17] described subgaleal drainage as a less invasive and safer option. Additionally, Gazzeri et al. [8] showed a significant association between ACT/ATT use and postoperative bleeding.

### Strengths and limitations

The strengths of this study are including all patients undergoing burr-hole drainage over a period of six years, two investigators independently assessing and discussing all cases, including all patients per hemisphere case and patient case according to the outcome measure, and assessing recurrence rate as well as reoperation rate. The study has limitations including its non-blinded and non-randomized nature, as well as being conducted at a single center with patients transferred from or transferred to secondary centers. First, differences in standard follow-up among hospitals may have influenced the results. The study might thus have underreported recurrences or systemic complications occurring in secondary centers. Second, the timing and bridging of ACT/ATT before surgery were not always clearly documented, particularly for transferred patients. The significant difference in the ability to taper ACT/ATT, favoring the subgaleal drain group, might have affected the results. A third limitation of the study is the high amount of missing data regarding smoking, which resulted in smoking being excluded from the multivariable analysis. Fourth, the small groups of patients with liver disease and kidney failure, makes it challenging to interpret the predictors of confounding in relation to these conditions. Another potential limitation is the significant difference in drain duration – drains were removed when productivity ceased. Subgaleal drains might have been removed earlier due to shorter productivity time. Differences in hospital length of stay could not be calculated due to many cases of patient discharge to reference centers. Finally, the choice of drain being based on surgeon preference introduced an inherent imbalance in the cohorts, with more cases receiving a subgaleal drain than a subdural drain. This imbalance could contribute to selection bias and potentially impact the generalizability of the findings. As such, the pseudo randomization was suboptimal. Our results therefore need to be confirmed in a prospective, truly randomized study. However, sample size calculation based on the method described by Rosner, indicates that 10,686 cases are required to achieve sufficient statistical power. Full power calculation is available in the supplementary data [21]. As many smaller studies have shown similar results, the necessity of such a large trial to ascertain the equipoise between these two techniques is doubtful.

## Conclusions

Based on the results of this retrospective single center cohort study, there is no clear superiority of subdural or subgaleal drain insertion concerning recurrence or reoperation. There is no supremacy of drain location regarding the risk of complications, morbidity or mortality. Based on these findings, the choice of drain insertion can remain at the discretion of the surgeon in our center. However, it is arguable that a large-scale, multicenter randomized controlled trial comparing both drain insertions could be conducted in the future.

## Supplementary Information

Below is the link to the electronic supplementary material.

Supplementary file1 (DOCX 44 KB)

## Data Availability

Data is available on request through the data manager of the UMC Utrecht.

## Abbreviations

**ACT**: Anticoagulant therapy
**ATT**: Antithrombotic therapy
**CI**: Confidence interval
**CSDH**: Chronic subdural hematoma
**CT**: Computed tomography
**DG**: Drain group
**mRS**: Modified Rankin Scale
**NA**: Not applicable
**NDG**: No drain group
**OR**: Odds ratio
**SD**: Standard deviation
**SDD**: Subdural drain
**SGD**: Subgaleal drain
