# Supplementary material for: Clinical outcome of subdural versus subgaleal drain after burr-hole drainage for chronic subdural hematoma
Source: Acta Neurochir (Wien). 2024 Nov 1;166(1):433. doi: 10.1007/s00701-024-06320-7 (PMC11527894; doi:10.1007/s00701-024-06320-7)
Supplement: Supplementary file 1 — Supplementary file1 (DOCX 44 KB) [file 701_2024_6320_MOESM1_ESM.docx]

**Table S1. Comparison of Recurrence between Subdural and Subgaleal Drain Groups receiving one burr hole**

|  | Subdural drain | Subgaleal drain | Odds Ratio (95% CI) | *p* value |
| --- | --- | --- | --- | --- |
| Recurrence | 6/49 (12.2) | 6/59 (10.2) | 0.81 (0.24-2.70) | 0.733 |
| Reoperation | 1/49 (2.0) | 6/59 (10.2) | 5.43 (0.63-46.8) | 0.123 |

*All data presented as n/N (%)*

*CI, confidence interval*

**Table S2. Power Calculation for Sample Size**

| **Sample Size** |  |
| --- | --- |
| Group 1 (SDD group) | 5343 |
| Group 2 (SGD group) | 5343 |
| Total | 10686 |

| **Study parameters** |  |
| --- | --- |
| Incidence, group 1 (SDD group) | 15.3% |
| Incidende, group 2 (SGD group) | 13.4% |
| Alpha | 0.05 |
| Beta | 0.2 |
| Power | 0.8 |


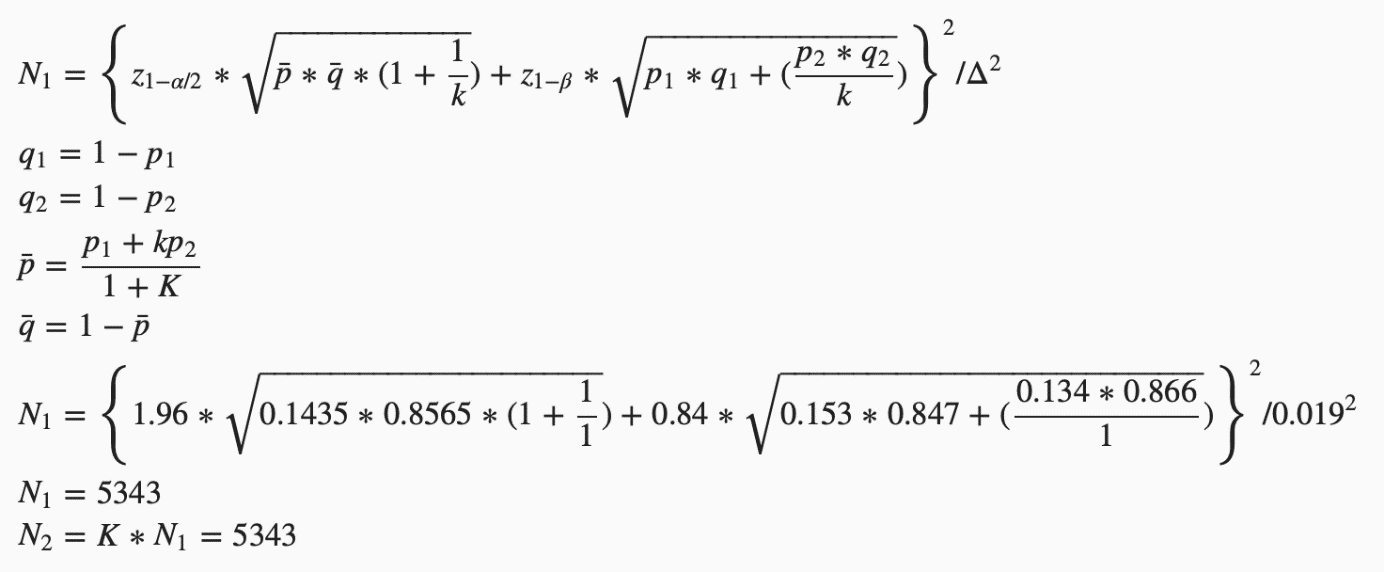


*p_1_, p_2_ = proportion (incidence) of groups #1 and #2*

*Δ = |p_2_-p_1_| = absolute difference between two proportions 
n_1_ = sample size for group #1 
n_2_ = sample size for group #2 
α = probability of type I error (usually 0.05) 
β = probability of type II error (usually 0.2) 
z = critical Z value for a given α or β 
K = ratio of sample size for group #2 to group #1*
